# Oral anticoagulants and concurrent rifampin administration in tuberculosis patients with non-valvular atrial fibrillation

**DOI:** 10.1186/s12872-023-03212-z

**Published:** 2023-04-04

**Authors:** Ki Won Hwang, Jin Hee Choi, Soo Yong Lee, Sang Hyun Lee, Min Ku Chon, Jungkuk Lee, Hasung Kim, Yong-Giun Kim, Hyung Oh Choi, Jeong Su Kim, Yong-Hyun Park, June Hong Kim, Kook Jin Chun, Gi-Byoung Nam, Kee-Joon Choi

**Affiliations:** 1 Division of Cardiology, Department of Internal Medicine, School of Medicine, Pusan National University, Pusan National University Yangsan Hospital, 20, Geumo-ro, Mulgeum-eup, Yangsan, Gyeongnam 626-770 South Korea; 2 Data Science Team, Hanmi Pharm. Co., Ltd, Seoul, Republic of Korea; 3 Division of Cardiology, Department of Internal Medicine, University of Ulsan College of Medicine, Ulsan, Republic of Korea; 4 Division of Cardiology, Department of Internal Medicine, Soonchunhyang University Hospital, Bucheon, Gyeonggi-do Republic of Korea; 5 Heart Institute, University of Ulsan College of Medicine, Asan Medical Center, Seoul, Republic of Korea

**Keywords:** Atrial fibrillation, Tuberculosis, Anticoagulation, Rifampin, Drug-drug interactions

## Abstract

**Background:**

Evidence and guidelines for Non-vitamin K antagonist oral anticoagulants (NOACs) use when prescribing concurrent rifampin for tuberculosis treatment in patients with non-valvular atrial fibrillation (NVAF) are limited.

**Methods:**

Using the Korean National Health Insurance Service database from January 2009 to December 2018, we performed a population-based retrospective cohort study to assess the net adverse clinical events (NACE), a composite of ischemic stroke or systemic embolism and major bleeding, of NOACs compared with warfarin among NVAF patients taking concurrent rifampin administration for tuberculosis treatment. After a propensity matching score (PSM) analysis, Cox proportional hazards regression was performed in matched cohorts to investigate the clinical outcomes.

**Results:**

Of the 735 consecutive patients selected, 465 (63.3%) received warfarin and 270 (36.7%) received NOACs. Among 254 pairs of patients after PSM, the crude incidence rate of NACE was 25.6 in NOAC group and 32.8 per 100 person-years in warfarin group. There was no significant difference between NOAC and warfarin use in NACE (hazard ratio [HR], 0.74; 95% confidence interval [CI], 0.48–1.14; *P* = 0.172). Major bleeding was the main driver of NACE, and NOAC use was associated with a statistically significantly lower risk of major bleeding than that with warfarin use (HR, 0.63; 95% CI, 0.40–1.00; *P* = 0.0499).

**Conclusions:**

In our population-based study, there was no statically significant difference in the occurrence of NACE between NOAC and warfarin use. NOAC use may be associated with a lower risk of major bleeding than that with warfarin use.

**Supplementary Information:**

The online version contains supplementary material available at 10.1186/s12872-023-03212-z.

## Background

Tuberculosis is a major health problem in South Korea. In 2019, the number of new tuberculosis cases in Korea was 23,821 (46.4 per 100,000), which was 9.9% lower than that in the previous year [1]. However, 47.1% of those new patients were 65 years or older. Tuberculosis infections are indolent and have a longer course of treatment compared with other viral or bacterial infections [2]. Patients with a tuberculosis infection also have an increased risk of thromboembolic events, such as deep vein thrombosis, pulmonary thromboembolism, or ischemic stroke [3–5]. Warfarin use is a clinically appropriate option of oral anticoagulants (OACs) in patients with concurrent rifampin administration [6]. However, rifampin is a potent inducer of hepatic cytochrome P450 (CYP450) 3A4 enzyme and transporter permeability glycoprotein (P-gp) system [7]. Concurrent rifampin administration impeded the anticoagulant effect of warfarin by enhancing CYP3A4 enzyme and jeopardized to achieve a therapeutic International Normalized Ratio (INR) value. Previous case reports described the difficulty to attain the optimal therapeutic range despite gradual increment of warfarin dose and episodes of bleeding following the withdrawal of rifampin [8–10].

Non-vitamin K antagonist oral anticoagulants (NOACs) are recommended as the preferred anticoagulation therapy for patients with non-valvular atrial fibrillation (NVAF) because they do not require frequent dosage adjustment of food restrictions to achieve target INR levels with strict laboratory monitoring [6,11]. Compared with warfarin, NOACs are presumed to have fewer food–drug and drug–drug interactions (DDIs). However, NOACs have the potential for clinically relevant drug interactions that are involved with either CYP enzyme and/or the P-gp transport system [12]. All NOACs are excreted by P-gp system. Apixaban and rivaroxaban are also metabolized by CYP3A4 enzyme. A strong P-gp inducers such as rifampin will decrease the NOAC exposure by > 50%. Reduced NOAC exposure could lead to an increased risk of thromboembolic events and worse efficacy [7]. There is a lack of studies specifically addressing the issue of NOAC use in NVAF patients with tuberculosis. Current data mainly focus on pharmacokinetic DDIs between NOACs and rifampin in healthy volunteers or on brief case reports in clinical practice [4, 13−16]. There is limited evidence on whether NOAC use is effective and safe in tuberculosis patients with concurrent rifampin administration. Thus, we aimed to evaluate the effectiveness and safety of NOACs compared with warfarin in NVAF patients who received a concurrent rifampin administration for tuberculosis treatment through a retrospective cohort study.

## Methods

### Data sources

This nationwide study is performed using the administrative claims datasets of the Korean National Health Insurance Service (NHIS). The NHIS provides a comprehensive health information that includes general specifications (age, sex, and region), diagnoses, laboratory tests, medical prescription recorders, treatment details, procedure, surgery, and the date of hospitalization in inpatient and outpatient service [17]. All diagnostic data are based on the International Classification of Disease, Tenth Revision (ICD-10). The study protocol was approved by the Institutional Review Board of Pusan National University Yangsan Hospital (IRB no. 05-2019-072).

### Study Population

**Fig. 1:**
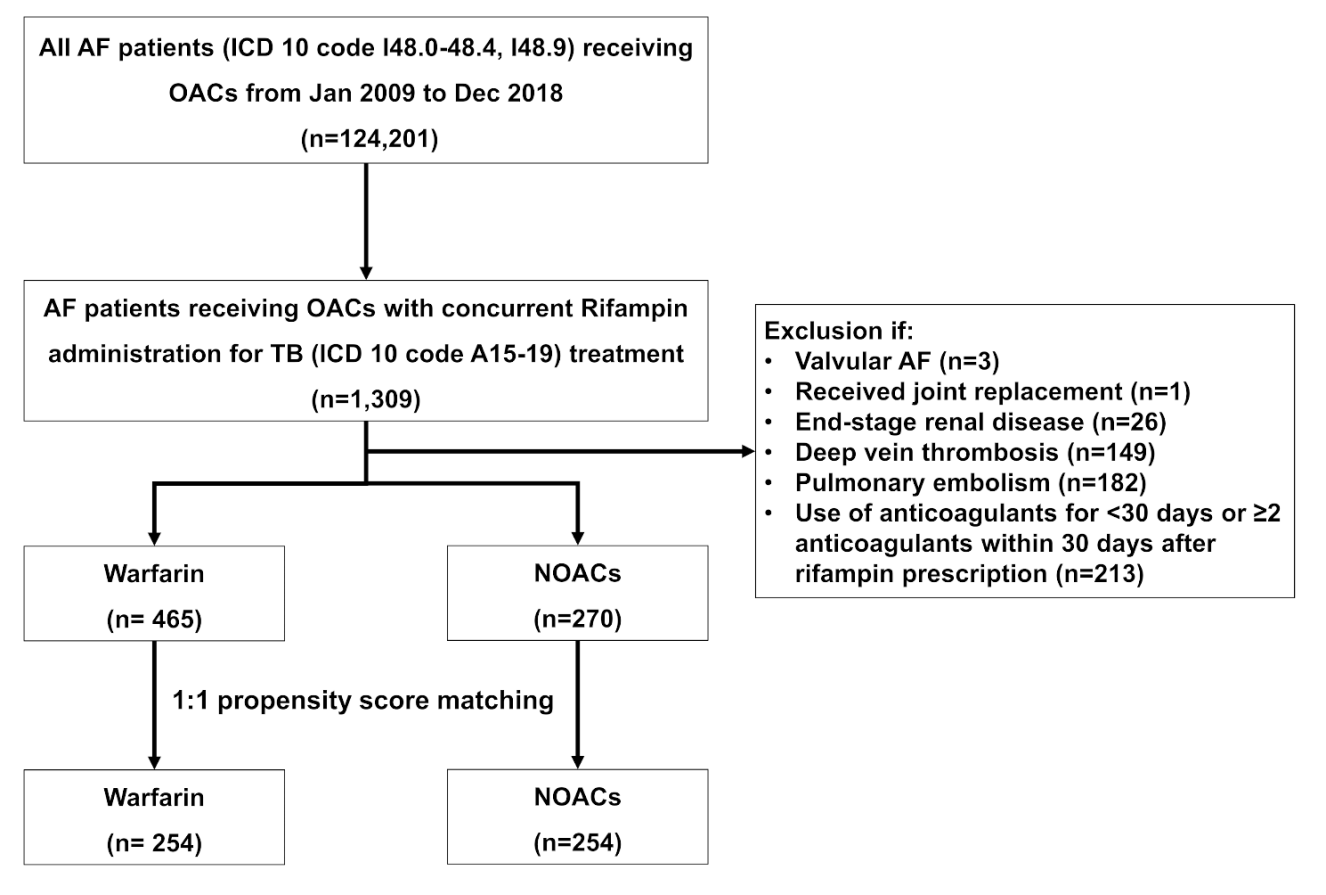
Enrollement flow of study patients. AF, atrial fibrillation; OAC, oral anticoagulant; TB, tuberculosis; NOAC, non–vitamin K antagonist oral anticoagulant

The study population included all patients with NVAF who visited medical institution(s) at least one or more with a primary diagnosis of tuberculosis coded as A15-19 according to the ICD-10 (see Supplementary Table S1 online) and received rifampin prescription between January 2009 and December 2018. To begin our patient selection process, we identified a population-based cohort of consecutive patients diagnosed with atrial fibrillation (AF) who received OACs. We then identified a subset of these patients who were prescribed a new rifampin for tuberculosis treatment. The date of the first dispensed rifampin was taken as the study index date. The exclusion criteria were as follows: Valvular AF (mitral stenosis or preexisting mechanical heart valve), withdrawal of OACs or ≥ 2 OACs within 30 days after the index medication, deep vein thrombosis, pulmonary thromboembolism or joint replacement surgery, all of which could be a potential alternative indication for OACs, and end-stage renal disease (Fig. 1).

### Clinical variables and Outcome Assessment

Detailed patient information, including demographics (age and sex), comorbidities (hypertension, diabetes, dyslipidemia, congestive heart failure [CHF], peripheral artery disease [PAD], chronic obstructive pulmonary disease [COPD], history of stroke/transient ischemic attack, ischemic heart disease, prior myocardial infarction [MI], and chronic kidney disease), and concurrent medications (antiplatelet agents, β-blockers, calcium-channel blockers, angiotensin-converting enzyme inhibitors or angiotensin receptor blockers, statins, and digoxin), was collected. Because not all eligible individuals received regular health screening program, missing data including body mass index or laboratory results were excluded from the analysis. We defined the overlap rifampin periods as the sum of prescription days with rifampin within 1 year from the first prescribed day.

The primary outcome was a comparison of net adverse clinical events (NACE), a composite of ischemic stroke or systemic embolism and major bleeding. The secondary outcomes included individual components from NACE, all-cause death, intracranial hemorrhage (ICH), and gastrointestinal (GI) bleeding. For outcome analyses, only events that occurred within 1 year after the index date were analyzed. Ischemic stroke was defined when the ICD-10 diagnosis code with hospitalization and concomitant brain imaging studies (computed tomography or magnetic resonance imaging) was identified [17]. Systemic embolism was defined as a hospital admission with the primary diagnosis code. Major bleeding was defined as a composite outcome of ICH or GI bleeding requiring hospitalization or bleeding that occurred at critical area or organ (intraspinal, intraocular, retroperitoneal, or intramuscular with compartment syndrome) [18].

### Statistical analysis

The comparisons between all continuous variables are presented as mean ± standard deviation. The descriptive variables are presented as absolute numbers and percentages of the total patients with the available data for each group or median (interquartile range [IQR]). Baseline characteristics were compared using Student’s *t*-test or the Mann–Whitney *U* test for continuous variables and Pearson’s chi-square test or Fisher’s exact test for descriptive variables.

For the comparisons, propensity score matching analysis was performed to balance the baseline covariates and reduce the residual bias between the NOAC and warfarin groups in NVAF patients with concurrent administration of rifampin. The propensity scores were estimated using a logistic regression model that incorporated all covariates that may be related to the outcome and/or treatment decisions. The covariates used for the propensity matching calculations were age, sex, comorbidities (CHA_2_DS_2_-VASc score, hypertension, diabetes mellitus, dyslipidemia, CHF, previous MI, PAD, or COPD), and concurrent medications. The propensity scores were estimated using a logistic regression model that incorporates all baseline covariates as listed in the Table 1. For propensity score matching, we used the one-to-one nearest-neighbor matching algorithm without replacements and adopted a caliper width equal to 0.25 of the standard deviation of the logit of the propensity score. We verified the performance of the propensity score model by comparing the distribution of covariates among the treatment groups before and after propensity score adjustment with the absolute standardized difference (ASD). Differences in baseline characteristics were evaluated by absolute standardized difference, and values with a negligible among different treatment groups were defined as ASD ≤ 0.1 (10%).

**Table 1:** Baseline Characteristics before and after propensity score matching

Characteristics	Propensity Score Matching					
	Before			After		
	NOACs (n = 270)	Warfarin (n = 465)	ASD	NOACs (n = 254)	Warfarin (n = 254)	ASD
Age, years	78.7 ± 8.8	76.1 ± 9.2	0.287	78.6 ± 8.8	78.3 ± 7.9	0.032
< 65	24 (8.9)	52 (11.2)		23 (9.1)	12 (4.7)	
65–74	35 (13.0)	119 (25.6)		32 (12.6)	55 (21.7)	
≥ 75	211 (78.1)	294 (63.2)		199 (78.3)	187 (73.6)	
Male gender –n (%)	154 (57.0)	277 (59.6)	0.050	147 (57.9)	142 (55.9)	0.040
CHA_2_DS_2_-VASc score	4.48 ± 1.55	4.01 ± 1.51	0.308	4.45 ± 1.42	4.45 ± 1.38	< 0.001
0–1	5 (1.9)	21 (4.5)		5 (2.0)	3 (1.2)	
2–3	56 (20.7)	146 (31.4)		54 (21.2)	62 (24.4)	
≥4	209 (77.4)	298 (64.1)		195 (76.8)	189 (74.4)	
Comorbidities –n (%)						
Congestive heart failure	166 (61.5)	260 (55.9)	0.116	153 (60.2)	152 (59.8)	0.008
Hypertension	228 (84.4)	384 (92.6)	0.050	212 (83.5)	220 (86.6)	0.088
Diabetes mellitus	85 (31.5)	151 (32.5)	0.020	82 (32.3)	84 (33.1)	0.017
Ischemic heart disease	176 (65.2)	297 (63.9)	0.034	166 (65.4)	170 (66.9)	0.033
Previous MI	35 (13.0)	60 (12.9)	0.002	33 (13.0)	38 (15.0)	0.057
Peripheral artery disease	137 (50.7)	141 (30.3)	0.425	123 (48.4)	106 (41.7)	0.135
prior stroke/TIA/SSE	27 (10.0)	51 (11.0)	0.031	26 (10.2)	29 (11.4)	0.038
COPD	189 (70.0)	257 (55.3)	0.308	175 (68.9)	178 (70.1)	0.026
Chronic kidney disease	35 (13.0)	69 (14.8)	0.022	35 (13.8)	38 (15.0)	0.034
Dyslipidemia	232 (85.9)	358 (77.0)	0.259	217 (85.4)	219 (86.2)	0.023
Concurrent Medication–n (%)						
Aspirin	116 (43.0)	192 (41.3)	0.035	106 (41.7)	107 (42.1)	0.008
P2Y_12_ inhibitor	54 (20.0)	79 (17.0)	0.079	48 (18.9)	51 (20.1)	0.030
Beta-blocker	136 (50.4)	234 (50.3)	0.007	125 (49.2)	133 (52.4)	0.063
Calcium-channel blocker	159 (58.9)	228 (49.0)	0.194	147 (57.9)	148 (58.3)	0.008
ACE inhibitor or ARB	162 (60.0)	291 (62.6)	0.051	154 (60.6)	155 (61.0)	0.008
Statin	111 (41.1)	201 (43.2)	0.037	106 (41.7)	113 (44.5)	0.056
Digoxin	61 (22.6)	168 (36.1)	0.309	60 (23.6)	62 (24.4)	0.018

Data are presented as mean ± standard deviation, or number (percentage)

NOAC, non-vitamin K antagonist oral anticoagulant; ASD, absolute standardized difference; CHA_2_DS_2_-VASc, congestive heart failure, hypertension, age ≥ 75 (doubled), diabetes, stroke (doubled), vascular disease, age 65–74, and sex (female); MI, myocardial infarction; TIA, transient ischemic accident; SSE, systemic embolism; COPD, chronic obstructive pulmonary disease; ACE, angiotensin converting enzyme; ARB, angiotensin II receptor blocker

Crude incidence rates were calculated as the number of events per 100 person-years for each clinical outcome. Cumulative incidence curves according to treatment groups were constructed using the Kaplan–Meier method with the log-rank test, and the univariate Cox proportional hazard model was used to compare NOACs with warfarin for the outcomes of interest. The results are expressed as hazard ratios (HRs) with 95% confidence intervals (CIs). Statistical significance was set at *P* < 0.05. All statistical calculations were performed with SAS Enterprise Guide version 7.1 (SAS Institute Inc., Cary, NC).

### Sensitivity analysis

First, sensitivity analysis was performed in all available patients without propensity score matching. In South Korea, the national health insurance system approved partial and full reimbursements of NOACs in January 2013 and July 2015, respectively [19]. Further, one-third of the study population was inevitably excluded after the propensity score matching. To assess the impact of drug interaction when rifampin was withdrawn, the period of concurrent rifampin administration was included in the sensitivity analysis after stratification to < 30 days versus ≥ 30 days. Univariate Cox proportional hazards regression models were used to identify the effectiveness and safety of NOACs compared with warfarin. A multivariate Cox proportional hazard regression analysis was performed with age, sex, CHA_2_DS_2_-VASc score, and all variables with *P* ≤ 0.1 in the univariate analyses. Second, we performed sensitivity analysis with restriction of the 6-month follow-up period because patients in the study only had the overlap period between rifampin and OAC for < 2 months.

## Results

### Baseline characteristics

**Fig. 2:**
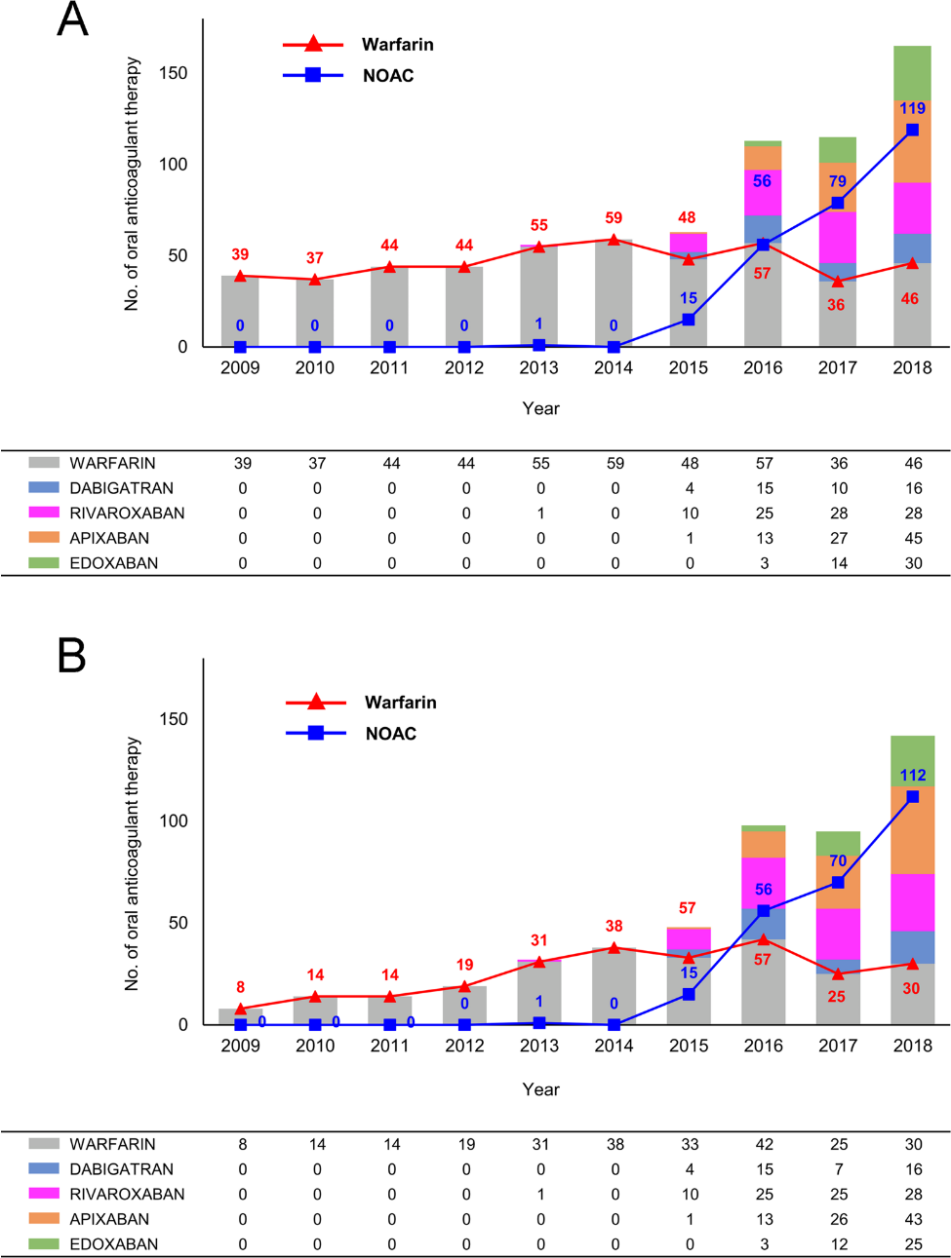
Trend in Non–vitamin K Antagonist Oral Anticoagulant (NOAC) and warfarin among patients with concurrent rifampin administration before (A) and after (B) propensity score matching

From January 2009 to December 2018, the initial cohort included 1,309 patients with AF who received OACs and concurrent rifampin administration for tuberculosis treatment. Among these, 735 patients were eligible for analysis; 465 (63.3%) were in the warfarin group and 270 (36.7%) were in the NOAC group (45 received dabigatran, 92 received rivaroxaban, 86 received apixaban, and 47 received edoxaban). Overall OAC use with concurrent rifampin administration increased rapidly from 39 patients in 2009 to 165 patients in 2018 (*P* for trend < 0.001) (Fig. 2A). There were sharp rise and diverging trends in OAC treatment from 2016, with year-on-year increase in NOAC use (56, 79, and 119, respectively) and a slight decrease in warfarin use (57, 36, and 46, respectively) over the study period. The proportion of concurrent rifampin and warfarin use declined over the study period (from 76.2% to 2015 to 27.9% in 2018; *P* for trend = 0.002).

Propensity score matching yielded 254 pairs of patients who were well-balanced with ASD ≤ 0.1 for all variables, except for a higher incidence of PAD in NOAC users (Table 1 and see Supplementary Fig. S1 online). Of the final NOAC cohort, 42 patients received dabigatran, 89 patients received rivaroxaban, 83 patients received apixaban, and 40 patients received edoxaban (Fig. 2B). The mean follow-up duration was shorter in the NOAC cohort than that in the warfarin cohort (7.0 ± 4.6 and 8.5 ± 4.6 months, *P* < 0.001). The mean duration of concurrent rifampin prescription was 40.0 ± 54.6 days (IQR, 12–43) in the NOAC users and 41.4 ± 72.7 days (IQR 10–36) in the warfarin users, with no significant difference between the two groups (*P* = 0.803).

### Net adverse clinical events in the Matched Cohort

**Fig. 3:**
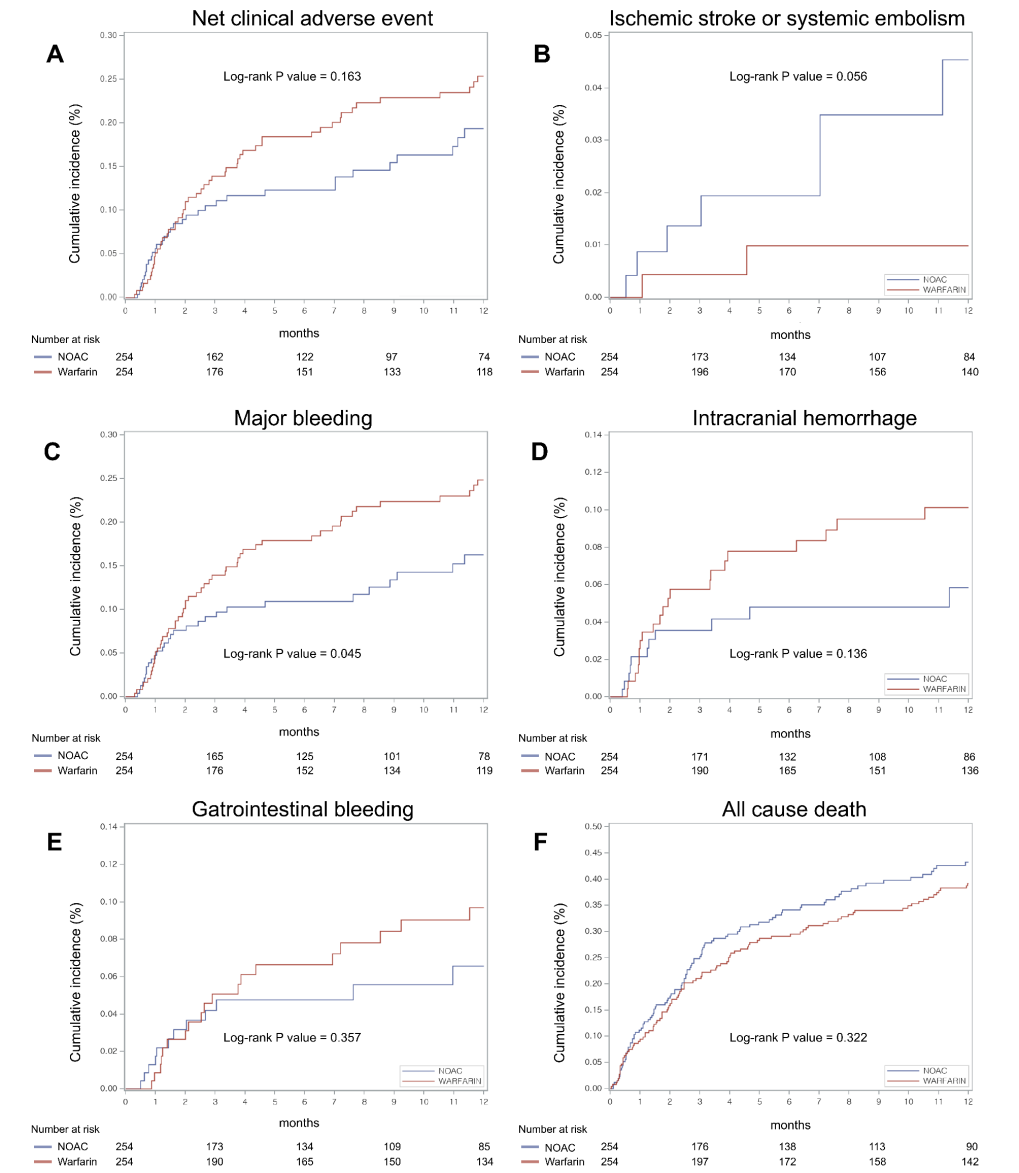
Cumulative Incidence Curves of Clinical Events Among the Propensity Score–Matched Cohort Using the Kaplan-Meier Method (A) Net adverse clinical events (B) Ischemic stroke or systemic embolism (C) Major bleeding (D) Intracranial hemorrhage (E) Gastrointestinal bleeding (F) All cause death

The incidence rates of each clinical outcome after propensity score matching and comparison between warfarin and NOAC use are summarized in Table 2. During follow-up, the crude incidence rate of NACE was 25.6 in NOAC users and 32.8 per 100 person-years in warfarin users, with no significant difference in the risk of NACE between the two groups (HR, 0.74; 95% CI, 0.48–1.14; *P* = 0.172; Fig. 3A). Major bleeding was the main driver of NACE, and the incidence rate of major bleeding was more common in warfarin users, with 32.0 per 100 person-years, as compared with 21.3 per 100 person-years in NOAC users (*P* = 0.045, Fig. 3C). NOAC use was associated with a 36% lower risk of major bleeding than warfarin use (HR, 0.63; 95% CI, 0.40–1.00; *P* = 0.0499). The difference in major bleeding was mainly driven by a reduction in both ICH and GI bleeding (Fig. 3D and E). There was a trend favoring NOAC compared with warfarin use, with no significant difference in ICH and GI bleeding events. There were numerically higher rates of ischemic stroke or systemic embolism in the NOAC use compared with warfarin, but they did not achieve statistical significance (4.9 and 1.1 per 100 person-years, respectively; HR, 3.53; 95% CI, 0.78–16.13; *P* = 0.102, Fig. 3B). Concurrent NOAC administration, as compared with warfarin, was associated with a similar risk of all-cause death (67.8 and 54.8 per 100 person-years, respectively; HR, 1.15; 95% CI, 0.87–1.53; *P* = 0.323, Fig. 3F).

**Table 2:** Clinical outcomes of Non–vitamin K Antagonist Oral Anticoagulants (NOACs) Use Compared With Warfarin Use After Propensity Score Matching

Outcome	NOACs		Warfarin		Hazard Ratio (95% CI)	P value
	*no. of patients with event*	*no. of 100* *patient-years*	*no. of patients with event*	*no. of 100* *patient-years*		
Net adverse clinical event	34	25.6	52	32.8	0.74 (0.48–1.14)	0.172
Ischemic stroke or systemic embolism	7	4.9	2	1.1	3.53 (0.78–16.13)	0.102
Major bleeding	29	21.3	51	32.0	0.63 (0.40-1.00)	0.0499
ICH	11	7.7	21	12.2	0.59 (0.28–1.22)	0.155
GI bleeding	12	8.4	19	11.0	0.72 (0.35–1.49)	0.381
All Cause death	100	67.8	98	54.8	1.15 (0.87–1.53)	0.323

ICH, intracranial hemorrhage; GI, gastrointestinal; CI, confidence interval

### Subgroup analysis

**Fig. 4:**
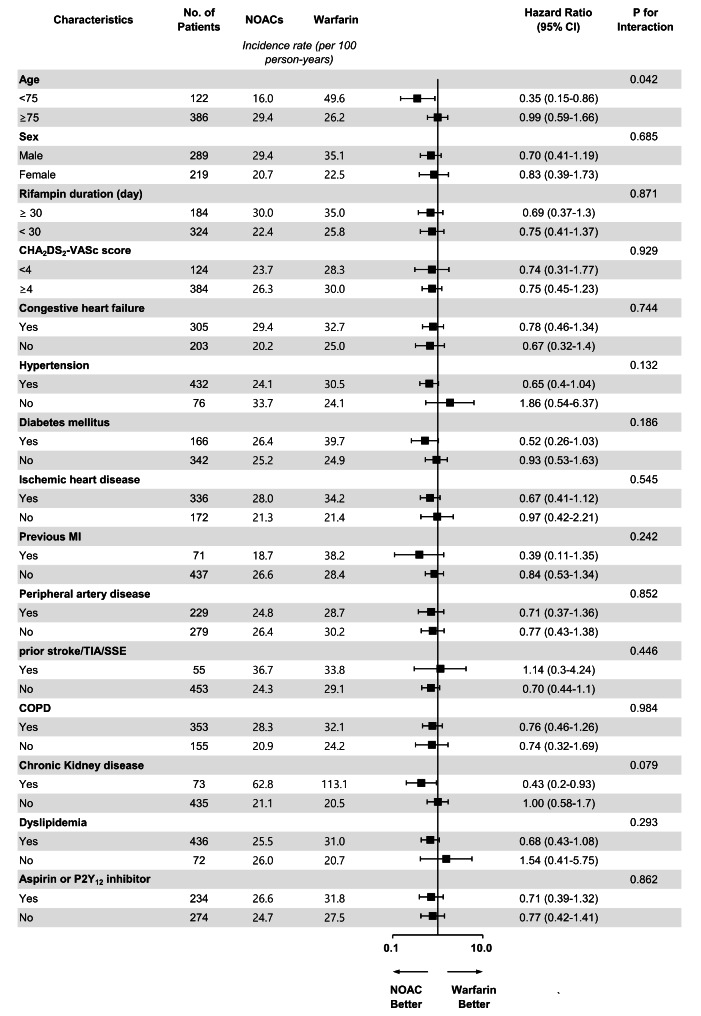
Risk of Net Adverse Clinical Events Associated With Non–vitamin K Antagonist Oral Anticoagulant (NOAC) Use Compared With Warfarin Use in Different Subgroups. MI, myocardial infarction; TIA, transient ischemic accident; SSE, systemic embolism; COPD, chronic obstructive pulmonary disease; CI, confidence interval

With respect to the NACE, subgroup analyses showed consistent results across multiple subgroups, with no significant interactions with baseline variables (Fig. 4). In patients < 75 years, there was a statistically significant lower rate of NACE with NOACs (HR, 0.35; 95% CI, 0.15–0.86; *P* for interaction = 0.042). There were significant interactions between OAC treatment and history of PAD for ischemic stroke or systemic embolism events (*P* = 0.0492; see Supplementary Table. S3 online).

### Sensitivity analysis

The results of the sensitivity analysis were generally consistent with those of the primary study analysis (see Supplementary Fig. S2 online). There was no significant between-group difference in the risk of net clinical adverse outcomes after adjustment (adjusted HR, 0.76; 95% CI, 0.51–1.13; *P* = 0.179). A multivariate Cox proportional analysis showed that, compared with warfarin users, there was a trend toward higher ischemic stroke or systemic embolism (adjusted HR, 2.92; 95% CI, 0.83–10.3; *P* = 0.094) and less major bleeding (adjusted HR, 0.66; 95% CI, 0.43–1.00; *P* = 0.052) in NOAC users.

For second sensitivity analysis with restriction of the 6-month follow-up period, HR trends for all clinical outcomes were generally in line with the main analysis (see Supplementary Table S2 online). Patients receiving NOAC had no difference in major bleeding compared with patients receiving warfarin (HR, 0.63; 95% CI, 0.37–1.05; *P* = 0.074).

## Discussion

In nationwide cohort study, we investigated the NACE of NOACs versus warfarin use in NVAF patients receiving concurrent rifampin administration for tuberculosis treatment. Our study yielded two major findings. (1) Overall OAC users with concurrent rifampin administration increased rapidly over the study period. Concurrent NOAC administration is responsible for the rapid increase; however, this combination therapy is not currently recommended in the guidelines. (2) NOAC use had a similar risk of NACE compared with warfarin in NVAF patients with concurrent rifampin administration for tuberculosis treatment. NOAC use was associated with a significantly lower risk of major bleeding, which was identified as the main driver of NACE, compared with warfarin. However, there was a trend toward a higher risk of ischemic stroke or systemic embolism events in NOAC when compared with warfarin use.

NOACs are now recommended as the preferred alternative to warfarin for reducing the risk of stroke associated with NVAF [6,11]. The therapeutic advantages of NOACs include a more rapid and predictable anticoagulant response, limited need for routine laboratory monitoring, and fewer food–drug interactions and DDIs compared to warfarin. However, physicians should have the recognition and understanding of the drug interaction when prescribing NOACs [6]. A substantial number of NOAC users received at least one potential co-medication such as CYP3A4 and/or P-gp inhibitor or inducer [20–22]. Previous studies mainly focused on the effect of major bleeding between NOACs and CYP3A4 and/or P-gp inhibitors [23,24]. Concurrent NOAC use with potent CYP3A4 and/or P-gp inducers was only listed as less than 1% of included patients in six European databases from five European countries [25]. Furthermore, they did not describe the impact between NOACs and potent CYP3A4 and/or P-gp inducers such as phenytoin, carbamazepine, and rifampin. In the analysis of the Taiwan National Health Insurance database, which evaluated 91,330 patients on a NOAC for NVAF, there were some NOACs use to recommend avoing combinations therapy by guideline [23]. Approximately 5% of NOAC users were prescribed potent inducers of P-gp and/or CYP3A4, such as rifampin or phenytoin. The authors reported that the combination of a NOAC with rifampin was associated with an increased risk of major bleeding compared with NOAC alone; however, they did not account for the efficacy and safety of NOAC use compared with warfarin in patients with concurrent rifampin administration. Our study reported that overall OAC users with concurrent rifampin administration seem to increase significantly during 10 years of the study periods, which was driven by the rapid and substantial increase in NOAC use since 2016. The proportion of overall OACs and NOACs in Korea accelerated since full reimbursement in July 2015. Despite clinically relevant drug interactions with NOACs, such combination therapy would be preferred or increased.

To the best of our knowledge, our study is the first to evaluate the clinically relevant effectiveness and safety of NOACs compared with warfarin in NVAF patients with concurrent rifampin administration for the treatment of tuberculosis. Based on the pharmacokinetic data resulting from NOACs use with concurrent rifampin administration in healthy volunteers, dabigatran exposure over a 7-day period resulted in a 67% reduction in the dabigatran area under the plasma concentration–time curve from zero to infinity [13,16]. In pharmacokinetic analysis of factor Xa inhibitors, co-administration of rifampin decreases apixaban exposure by up to 54%, rivaroxaban by up to 50%, and edoxaban by up to 35% [12–15]. NOAC use should be avoided or used with great caution and careful monitoring because rifampin reduces NOAC plasma concentration. Therefore, anticoagulant therapy with warfarin is recommended for patients with concurrent rifampin administration [6]. However, when rifampin is initiated or discontinued for the treatment of tuberculosis, frequent INR monitoring and appropriate adjustment of warfarin dosage are needed to minimize the risk of ischemic stroke and bleeding. NOACs have major advantages over warfarin, including a wide therapeutic window and low inter- or intra-individual variations. Furthermore, in patients on warfarin with low time in therapeutic range < 70%, switch to a NOAC was recommended [26]. In the present study, NOACs with rifampin co-administration are likely to have clinically relevant outcomes compared with warfarin, despite inappropriate use with potential DDIs. Although the anticoagulant effect of NOAC depends on drug exposure, the relationship between plasma concentration and clinical outcomes is more complex [13]. Plasma NOAC concentration is associated with the risk of clinical outcomes, particularly a steeper slope in major bleeding compared with ischemic stroke or systemic embolism. Inhibition of endogenous factor Xa activity yielded a plateau when the NOAC plasma concentration was above a constant level. Data from the ENGAGE AF-TIMI 48 showed that the increase in edoxaban plasma concentration was inversely correlated with the endogenous factor Xa activity [27]. Patients randomized to a lower dose edoxaban regimen (30/15 mg) had a significantly lower risk of net clinical outcome than those randomized to higher dose edoxaban regimen (60/30 mg), and the difference was primarily driven by a 36% decrease in major bleeding in those given a lower dose edoxaban regimen (1.82% vs. 2.87%; HR, 0.64; 95% CI, 0.55–0.74; *P* < 0.001) [28]. In Taiwan registry [29], 27% of patients received off-label reduced doses of NOACs. Compared with warfarin, this underdosing was associated with a higher risk of stroke or systemic embolism and a lower risk of major bleeding. However, INR data was not available on a Taiwan population cohort. The poor quality of warfarin therapy correlates with adverse clinical outcomes [26]. Lee et al [30]. reported that well-controlled warfarin is as safe as, and more effective than underdosed NOACs. Although the reasons for this underdosing remain uncertain, those receiving underdosed NOACs were older, more likely female, had a higher CHA_2_DS_2_-VASc score, and had concomitant use of an antiplatelet drug other than warfarin. The comorbidities in patients with underdosed NOACs was associated that rates of major bleeding were not reduced compared with warfarin.

Our study reported that concomitant aspirin and P2Y_12_ inhibitors were used in 41.9% and 18.1% of patients, respectively. Other studies using the Korean NHIS database have reported lower rates of concomitant antiplatelet agent use [31]. The mean age of our cohort was older, and the proportion of patients with ischemic heart disease and PAD was higher in our study. The risk factors for tuberculosis infection include medical conditions (such as alcohol abuse and diabetes), medical treatments (such as corticosteroids and organ transplants), and lower body weight [3]. In addition to pharmacokinetic interaction, the combination therapy with antiplatelet agents can influence the efficacy and safety of both warfarin and NOACs, owing to their pharmacodynamic effects on blood coagulation and platelet aggregation [32]. In a meta-analysis of phase III trials [33], approximately 33% of patients were prescribed concomitant use of antiplatelet agents and OACs, which were associated with greater risks of major bleeding and with no benefit for reduction in stroke. The risk of major bleeding was lower on dabigatran 110 mg and lower-dose edoxaban (30/15 mg) than on warfarin in patients with concomitant antiplatelet prescription [34,35]. We observed similar effects of NOACs compared with warfarin on net clinical benefit irrespective of concomitant antiplatelet agent.

### Limitations

Our study had several limitations. First, our results are based on the Korean NHIS claims database, which generates data for reimbursement rather than for research. Therefore, there are some unmeasurable confounding factors, including physicians’ decisions and detailed laboratory findings such as creatinine clearance or INR control in warfarin-treated patients. The lack of INR values was an inherent limitation in our retrospective study using claims databases, which could potentially influence the estimated treatment effects of NOACs in comparison to warfarin. During concomitant rifampin prescription, it was difficult to maintain a therapeutic INR level in patients receiving warfarin [36]. This impact could have contributed to the similar effectiveness and safety of NOACs versus warfarin in our study. And, detailed NOAC dosage information was not analyzed in the present study. Second, the study population might be too relatively small to have the sufficient statistical power of the analysis. Third, our rationale for the rifampin and NOAC combination therapy has been anchored on the assumption that the dissipation of CYP induction after the rifampin discontinuation occurs gradually [9], and the reduced exposure to NOACs was observed up to several weeks or months after rifampin discontinuation. In this study, the cumulative event curves for NACE started to diverge around 1 month. These findings were consistent over a period of 1 year of follow-up. However, the mean duration of concomitant administration between rifampin and OACs was 40.7 ± 64.3 days. We did not identify the duration of rifampin use before OACs were prescribed. And patients were not censored by rifampin discontinuation while OACs are co-administered. Adverse events might be occurred when OACs and rifampin were not both prescribed. Although our results remained consistent in the sensitivity analyses with restriction of the 6 month follow-up period, NOAC use could be considered in patients with a short remaining term of rifampin or labile INR level. Therefore, our study might not represent sufficient evidence that it is effective and well tolerated in a long-term treatment strategy for 6 months using rifampin and NOAC combination. Further well-designed, randomized controlled trials are needed to confirm our results.

## Conclusions

Overall OAC users with concurrent rifampin administration increased rapidly over the study period, and it was mainly associated with a sharp increase in NOAC prescription since full reimbursement in Korea, although this combination therapy is not recommended in the current guidelines. There was no statistically significant difference in the occurrence of NACE, a composite of ischemic stroke or systemic embolism and major bleeding, between NOAC and warfarin in NAVF patients who received concurrent rifampin administration for the treatment of tuberculosis. NOAC use may be associated with reduced risk of major bleeding, but not significantly increased risk of ischemic stroke or systemic embolism. The use of NOACs needs to be carefully considered in tuberculosis patients with poor INR control.

## Electronic supplementary material

Below is the link to the electronic supplementary material.

Additional File: All supplementary tables and figures.

## Data Availability

The data that support the findings of this study are available from the Korea National Health Insurance Service but the access to data is only available for the researchers who have applied for and have been licensed. Further information is available in online homepage of National Health Insurance Sharing Service (https://nhiss.nhis.or.kr).

## Abbreviations

OAC: Oral anticoagulant
CYP: Cytochrome
INR: International Normalized Ratio
NOAC: Non-vitamin K antagonist oral anticoagulant
NVAF: Non-valvular atrial fibrillation
DDI: Drug–drug interaction
NHIS: National Health Insurance Service
ICD-10: International Classification of Disease, Tenth Revision
AF: Atrial fibrillation
CHF: Congestive heart failure
PAD: Peripheral artery disease
COPD: Chronic obstructive pulmonary disease
MI: Myocardial infarction
NACE: Net adverse clinical event
ICH: Intracranial hemorrhage
GI: Gastrointestinal
IQR: Interquartile range
ASD: Absolute standardized difference
HR: Hazard ratio
CI: Confidence interval
