# Supplementary material for: Oral anticoagulants and concurrent rifampin administration in tuberculosis patients with non-valvular atrial fibrillation
Source: BMC Cardiovasc Disord. 2023 Apr 4;23:182. doi: 10.1186/s12872-023-03212-z (PMC10074893; doi:10.1186/s12872-023-03212-z)
Supplement: Supplementary file 1 — Additional File: All supplementary tables and figures. [file 12872_2023_3212_MOESM1_ESM.docx]

Supplementary Table 1. Definitions of comorbidities and clinical outcome according to ICD-10 codes

| Disease | ICD-10 codes (1) | Additional definition (2) | Combination |
| --- | --- | --- | --- |
| Atrial fibrillation | I48.0-I48.4, I48.9 | Admission ≥ 1 or outpatient department ≥ 2 | 1+2 |
| Mitral stenosis * | I05.0 I05.2 | Or claim code for open  commissurotomy or  percutaneous valvuloplasty | 1 or 2 |
| Mechanical valve* | Z95.2–Z95.4 | Or claim code for surgical valve replacement | 1 or 2 |
| Received joint replacement* | N0711, N1711, N1721, N2070, N3710, N3721, N3717, N3720, N2072, N2077, N3722, N3727 | Admission ≥ 1 | 1+2 |
| End-stage renal disease* | I185, Z49 | Dialysis ≥2 | 1+2 |
| Deep vein thrombosis * | I80.2 | Admission ≥ 1 | 1+2 |
| Pulmonary embolism * | I26 | Admission ≥ 1 | 1+2 |
| Congestive heart failure | I50 | Admission ≥ 1 or outpatient department ≥ 1 | 1+2 |
| Hypertension | I10–I13, I15 and minimum 1 prescription of anti-hypertensive drug | Admission ≥ 1 or outpatient department ≥ 2 | 1+2 |
| Diabetes | E10–E14, and minimum 1 prescription of anti-diabetic drugs | Admission ≥ 1 or outpatient department ≥ 2 | 1+2 |
| Dyslipidemia | E78 | Admission ≥ 1 or outpatient department ≥ 2 | 1+2 |
| COPD | J41-44 | Admission ≥ 1 or outpatient department ≥ 1 |  |
| Previous myocardial infarction | I21–I22 | Admission ≥ 1 or outpatient department ≥ 1 | 1+2 |
| Ischemic heart disease | I20-I25 |  | 1 |
| Peripheral artery disease | I70.0, I70.1, I70.2, I70.8, I70.9 | Admission ≥ 1 or outpatient department ≥ 2 | 1+2 |
| Chronic kidney disease | N18, N19 | Admission ≥ 1 or outpatient department ≥ 2 | 1+2 |
| Transient ischemic attack | G45 | Admission ≥ 1 or outpatient department ≥ 1 | 1+2 |
| Previous Ischemic stroke | I63, I64 | With admission and brain imaging (CT or MRI) | 1+2 |
| Systemic embolism† | I74 | With admission | 1+2 |
| Ischemic stroke† | I63, I64 | With admission and brain imaging (CT or MRI) | 1+2 |
| Extracranial or unclassified major bleeding† | D62, H05.2, H35.6, H43.1,  J94.2, M25.0, R04.2 |  | 1 |
| Gastrointestinal bleeding† | I85.0, K22.1, K22.8, K25.0, K25.2, K25.4, K25.6, K26.0, K26.2, K26.4, K26.6, K27.0, K27.2, K27.4, K27.6, K28.0, K28.2, K28.4, K28.6, K29.0, K31.8, K55.2, K57.0, K57.1, K57.2, K57.3, K57.4, K57.5, K57.8, K57.9, K62.5, K66.1 K92.0, K92.1, K92.2 | Hospitalization and RBC ≥1 pack | 1+2 |
| Intracranial hemorrhage† | I60–I62 | With hospitalization and  brain imaging (CT or MRI) | 1+2 |

COPD, chronic obstructive pulmonary disease; RBC, red blood cell

* Used in exclusion criteria, †Used in clinical outcome

Supplementary Figure 1. Plot of absolute standardized mean differences, before and after propensity score matching, for the assessment of the Clinical outcomes. MI, myocardial infarction; COPD, chronic obstructive pulmonary disease; ACE, angiotensin converting enzyme; ARB, angiotensin II receptor blocker


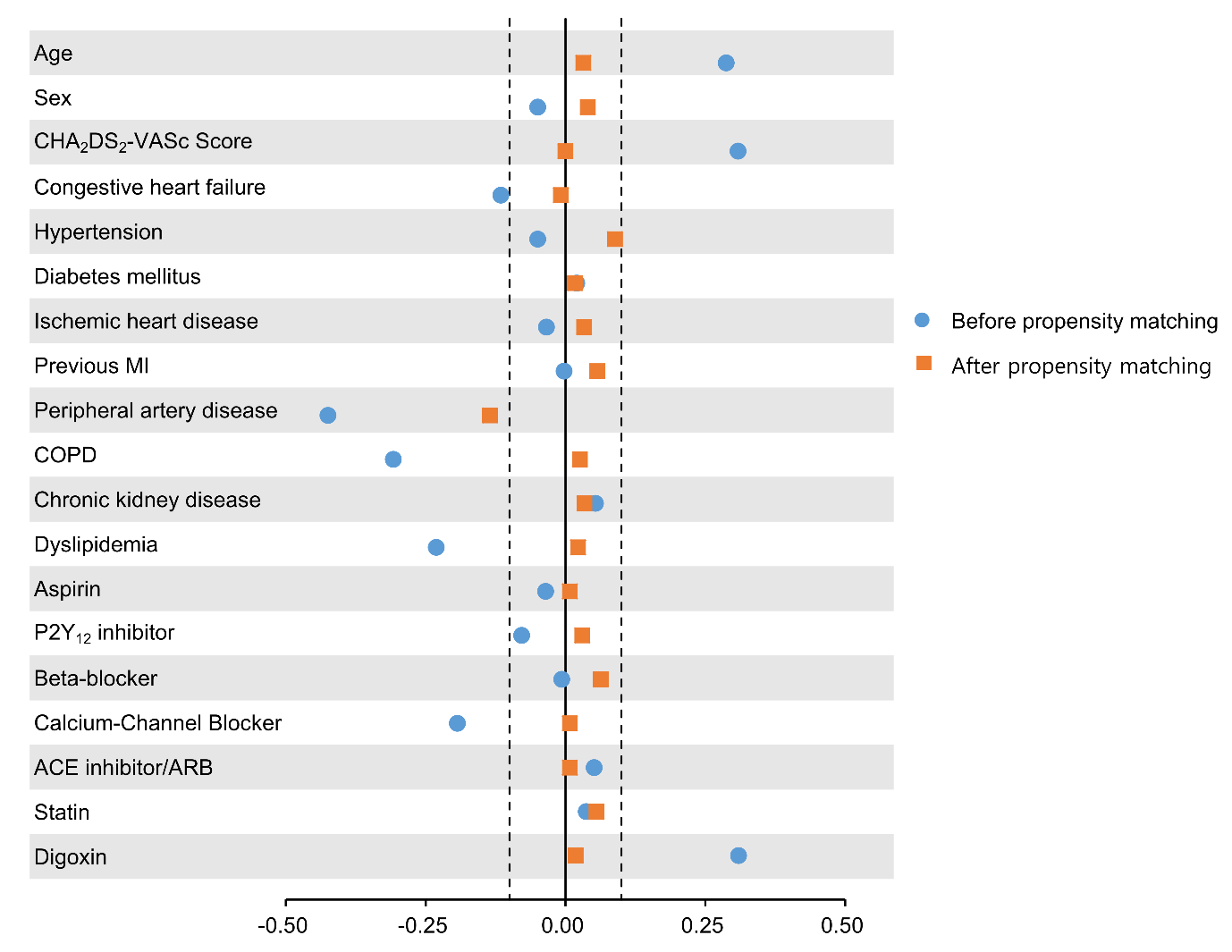


Supplementary Figure 2. Forest plot of the Clinical outcomes between Non–vitamin K Antagonist Oral Anticoagulant (NOAC) Use and Warfarin Use after multivariate adjustment for sensitivity analyses. Adjusted hazard ratios were adjusted for the age, sex, CHA_2_DS_2_-VASc score, Comorbidities (congestive heart failure, hypertension, diabetes mellitus, ischemic heart disease, previous MI, peripheral artery disease, prior stroke/TIA/SSE, COPD, chronic kidney diease and dyslipidemia), Concurrent Medication (aspirin, P2Y_12_ inhibitor, beta-blocker, calcium-channel blocker, ACE inhibitor or ARB, statin and digoxin) and overlap rifampin period (< 30 days versus ≥ 30 days). Intracranial hemorrhage, ICH; Gastrointestinal, GI; CI, confidence interval; MI, myocardial infarction; TIA, transient ischemic accident; SSE, systemic embolism; COPD, chronic obstructive pulmonary disease; ACE, angiotensin converting enzyme; ARB, angiotensin II receptor blocker


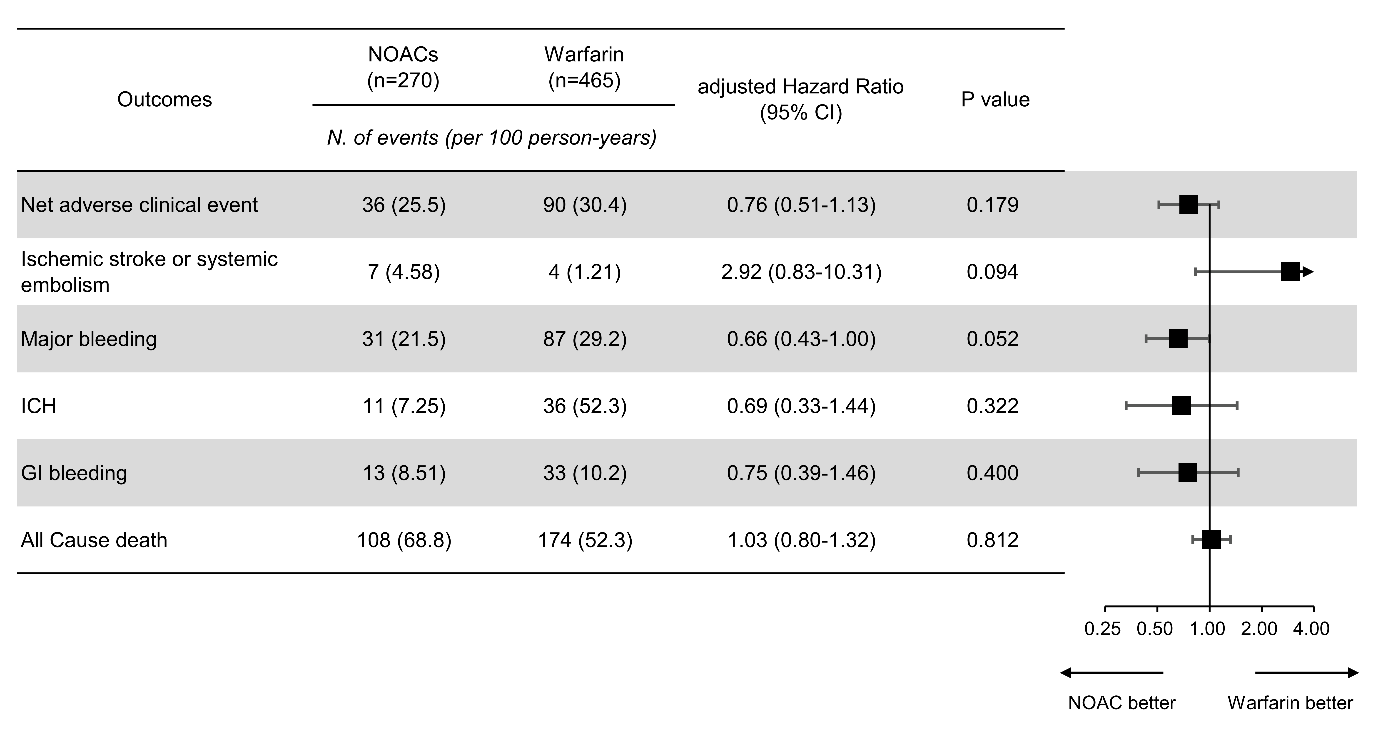


Supplementary Table 2. Hazard ratios of the Clinical outcomes between Non–vitamin K Antagonist Oral Anticoagulant (NOAC) Use and Warfarin Use for sensitivity analysis with restriction of the 6-month follow-up period. Intracranial hemorrhage, ICH; Gastrointestinal, GI; HR, Hazard Ratio; CI, confidence interval

| Outcome | NOACs | Warfarin | HR (95% CI) | P value |
| --- | --- | --- | --- | --- |
|  | N. of events (per 100 person-years) | |  |  |
| Net adverse clinical event | 26 (30.9) | 40 (43.4) | 0.70 (0.43-1.14) | 0.151 |
| Ischemic stroke or systemic embolism | 4 (4.5) | 2 (2.0) | 1.95 (0.37-10.34) | 0.435 |
| Major bleeding | 23 (15.5) | 39 (23.2) | 0.63 (0.37-1.05) | 0.074 |
| ICH | 10 (11.3) | 17 (17.5) | 0.64 (0.29-1.39) | 0.256 |
| GI bleeding | 10 (11.2) | 14 (14.3) | 0.78 (0.34-1.74) | 0.537 |
| All Cause death | 82 (89.8) | 73 (73.6) | 1.2 (0.88-1.65) | 0.257 |

Supplementary Table 3. Hazard ratios for secondary outcomes according subgroups in warfarin and Non–vitamin K Antagonist Oral Anticoagulant (reference).

|  | **Ischemic stroke or SSE** | **Major bleeding** | **ICH** | **GI bleeding** | **All Cause death** |
| --- | --- | --- | --- | --- | --- |
|  | HR  (95% CI) | HR  (95% CI) | HR  (95% CI) | HR  (95% CI) | HR  (95% CI) |
| **Age** | P*=0.6827 | P*=0.0343 | P*=0.1387 | P*=0.2776 | P*=0.0208 |
| <75 | 2.304  (0.222-23.810) | 0.257  (0.091-0.724) | 0.198  (0.032-1.203) | 0.389  (0.089-1.706) | 0.460  (0.197-1.072) |
| ≥75 | 3.817  (0.542-27.027) | 0.875  (0.514-1.488) | 0.898  (0.387-2.079) | 0.947  (0.401-2.232) | 1.290  (0.951-1.751) |
| **Sex** | P*=0.3214 | P*=0.6449 | P*=0.8198 | P*=0.8653 | P*=0.5145 |
| Male | 11.111  (0.419-333.33) | 0.590  (0.337-1.031) | 0.554  (0.225-1.368) | 0.709  (0.294-1.709) | 1.242  (0.859-1.795) |
| Female | 1.597  (0.270-9.434) | 0.727  (0.330-1.600) | 0.690  (0.203-2.342) | 0.772  (0.219-2.725) | 1.033  (0.669-1.592) |
| **Rifampin duration (day)** | P*=0.5945 | P*=0.8334 | P*=0.7968 | P*=0.4568 | P*=0.550 |
| ≥ 30 | 2.320  (0.467-11.494) | 0.590  (0.303-1.147) | 0.637  (0.247-1.645) | 0.526  (0.195-1.416) | 1.376  (0.841-2.252) |
| < 30 | 6.250  (0.150-250.00) | 0.655  (0.350-1.225) | 0.507  (0.162-1.592)) | 0.985  (0.342-2.841) | 1.075  (0.763-1.515) |
| **CHA_2_DS_2_-VASc score** | P*=0.4005 | P*=0.4276 | P*=0.297 | P*=0.8818 | P*=0.953 |
| ≤3 | 10.989  (0.351-333.33) | 0.456  (0.166-1.250) | 0.134  (0.005-3.521) | 0.688  (0.174-2.710) | 1.151  (0.608-2.179) |
| >3 | 1.934  (0.364-10.309) | 0.704  (0.421-1.178) | 0.703  (0.330-1.502) | 0.763  (0.326-1.786) | 1.136  (0.831-1.553) |
| **Congestive heart failure** | P*=0.622 | P*=0.956 | P*=0.3289 | P*=0.2335 | P*=0.9099 |
| Yes | 4.065  (0.577-28.517) | 0.635  (0.361-1.116) | 0.762  (0.326-1.783) | 0.495  (0.176-1.389) | 1.171  (0.819-1.672) |
| No | 2.188  (0.210-22.321) | 0.635  (0.293-1.374) | 0.331  (0.075-1.453) | 1.129  (0.395-3.226) | 1.117  (0.710-1.761) |
| **Hypertension** | P*=0.9736 | P*=0.161 | P*=0.1719 | P*=0.4198 | P*=0.2243 |
| Yes | 3.145  (0.668-14.706) | 0.557  (0.339-0.917) | 0.497  (0.229-1.081) | 0.661  (0.305-1.429) | 1.080  (0.799-1.460) |
| No | 2.538  (0.026-250.00) | 1.582  (0.444-5.650) | 7.752  (0.183-333.33) | 1.420  (0.137-14.706) | 1.751  (0.781-3.937) |
| **Diabetes** | P*=0.0533 | P*=0.5636 | P*=0.501 | P*=0.2721 | P*=0.0173 |
| Yes | 0.197  (0.005-8.130) | 0.543  (0.270-1.088) | 0.713  (0.292-1.742) | 0.415  (0.113-1.524) | 0.696  (0.419-1.156) |
| No | 19.608  (0.913-500.00) | 0.705  (0.386-1.289) | 0.426  (0.119-1.529) | 0.984  (0.407-2.381) | 1.456  (1.034-2.049) |
| **Ischemic heart disease** | P*=0.6374 | P*=0.4854 | P*=0.9335 | P*=0.6094 | P*=0.1247 |
| Yes | 4.292  (0.608-30.303) | 0.567  (0.330-0.974) | 0.584  (0.261-1.307) | 0.647  (0.272-1.538) | 0.987  (0.704-1.385) |
| No | 1.957  (0.188-20.408) | 0.870  (0.371-2.041) | 0.669  (0.125-3.571) | 0.966  (0.260-3.597) | 1.618  (0.972-2.688) |
| **Previous MI** | P*=0.6501 | P*=0.3596 | P*=0.3316 | P*=0.9894 | P*=0.327 |
| Yes | N/A | 0.385  (0.110-1.353) | 0.269  (0.040-1.795) | 0.701  (0.068-7.246) | 0.836  (0.422-1.656) |
| No | 3.521  (0.775-15.873) | 0.708  (0.433-1.159) | 0.739  (0.332-1.645) | 0.733  (0.343-1.565) | 1.236  (0.907-1.684) |
| **Peripheral artery disease** | P*=0.0492 | P*=0.5062 | P*=0.364 | P*=0.8373 | P*=0.5211 |
| Yes | 0.185  (0.004-7.634) | 0.747  (0.389-1.435) | 0.734  (0.309-1.745) | 0.819  (0.249-2.695) | 1.289  (0.835-1.988) |
| No | 20.833  (0.970-500.00) | 0.542  (0.283-1.036) | 0.321  (0.075-1.376) | 0.707  (0.283-1.764) | 1.073  (0.739-1.558) |
| **prior stroke/TIA/SSE** | P*=0.2552 | P*=0.9933 | P*=0.9515 | P*=0.413 | P*=0.1625 |
| Yes | 1.209  (0.203-7.194) | 0.649  (0.109-3.846) | 0.528  (0.051-5.495) | 2.994  (0.032-250.00) | 0.732  (0.346-1.548) |
| No | 10.989  (0.415-333.33) | 0.642  (0.400-1.029) | 0.601  (0.280-1.290) | 0.682  (0.325-1.431) | 1.241  (0.916-1.681) |
| **COPD** | P*=0.9704 | 0.9748 | P*=0.2925 | P*=0.5716 | P*=0.0286 |
| Yes | 2.857  (0.335-24.390) | 0.656  (0.386-1.116) | 0.760  (0.345-1.675) | 0.643  (0.260-1.587) | 1.399  (1.013-1.931) |
| No | 3.058  (0.403-23.256) | 0.635  (0.260-1.553) | 0.252  (0.036-1.773) | 0.957  (0.277-3.311) | 0.673  (0.376-1.205) |
| **Chronic Kidney disease** | P*=0.9324 | P*=0.1756 | P*=0.0702 | P*=0.2844 | P*=0.1084 |
| Yes | 3.413  (0.037-333.33) | 0.417  (0.194-0.898) | 0.384  (0.154-0.957) | 0.372  (0.082-1.684) | 0.722  (0.372-1.401) |
| No | 3.086  (0.654-14.493) | 0.810  (0.456-1.439) | 1.855  (0.446-7.752) | 0.947  (0.409-2.198) | 1.280  (0.937-1.751) |
| **Dyslipidemia** | P*=0.9418 | P*=0.368 | P*=0.7493 | P*=0.3461 | P*=0.4579 |
| Yes | 3.021  (0.642-14.286) | 0.589  (0.363-0.956) | 0.617  (0.296-1.285) | 0.645  (0.298-1.397) | 1.106  (0.817-1.497) |
| No | 3.937  (0.043-333.33) | 1.215  (0.302-4.902) | 0.474  (0.005-47.619) | 1.761  (0.170-18.182) | 1.441  (0.681-3.049) |
| **Aspirin or P2Y_12_ inhibitor** | P*=0.5088 | P*=0.6979 | P*=0.307 | P*=0.4501 | P*=0.1961 |
| Yes | 8.197  (0.266-250.00) | 0.582  (0.305-1.114) | 0.447  (0.175-1.142) | 0.961  (0.348-2.653) | 0.949  (0.633-1.422) |
| No | 2.119  (0.398-11.236) | 0.698  (0.368-1.326) | 0.946  (0.288-3.106) | 0.558  (0.195-1.592) | 1.376  (0.931-2.033) |

*P for interaction

SSE, systemic embolism; ICH, intracranial hemorrhage; GI, gastrointestinal; HR, hazard ratio; CI, confidence interval; CHA_2_DS_2_-VASc, congestive heart failure, hypertension, age ≥75 (doubled), diabetes, stroke (doubled), vascular disease, age 65-74, and sex (female); MI, myocardial infarction; TIA, transient ischemic accident; COPD, chronic obstructive pulmonary disease
